# An Optimal Animal Model of Ischemic Stroke Established by Digital Subtraction Angiography-Guided Autologous Thrombi in Cynomolgus Monkeys

**DOI:** 10.3389/fneur.2022.864954

**Published:** 2022-04-25

**Authors:** Juan Ye, Hailong Shang, Hongdi Du, Ying Cao, Lei Hua, Feng Zhu, Wei Liu, Ying Wang, Siyu Chen, Zhifu Qiu, Hailin Shen

**Affiliations:** ^1^Department of Radiology, Suzhou Kowloon Hospital, Shanghai Jiaotong University School of Medicine, Suzhou, China; ^2^Department of Radiotherapy, Suzhou Kowloon Hospital, Shanghai Jiaotong University School of Medicine, Suzhou, China; ^3^Department of Pharmacology, Prisys Biotechnologies Co., Ltd., Shanghai, China; ^4^Department of Endocrinology, Dushu Lake Hospital Affiliated to Soochow University, Suzhou, China

**Keywords:** DSA-guided, MCAO, CT, MRI, animal model

## Abstract

**Objective:**

Ischemic stroke seriously threatens human health, characterized by the high rates of incidence, disability, and death. Developing a reliable animal model that mimics most of the features of stroke is critical for pathological studies and clinical research. In this study, we aimed to establish and examine a model of middle cerebral artery occlusion (MCAO) guided by digital subtraction angiography (DSA) in cynomolgus monkeys.

**Materials and Methods:**

In this study, 15 adult male cynomolgus monkeys were enrolled. Under the guidance of DSA, a MCAO model was established by injecting an autologous venous clot into the middle cerebral artery (MCA) *via* femoral artery catheter. Thrombolytic therapy with alteplase (rt-PA) was given to eight of these monkeys at 3 h after the occlusion. Blood test and imaging examination, such as computed tomography angiography (CTA), CT perfusion (CTP), brain magnetic resonance imaging (MRI), and brain magnetic resonance angiography (MRA), were performed after the operation to identify the post-infarction changes. The behavioral performance of cynomolgus monkeys was continuously observed for 7 days after operation. The animals were eunthanized on the 8th day after operation, and then the brain tissues of monkeys were taken for triphenyltetrazolium chloride (TTC) staining.

**Results:**

Among the 15 cynomolgus monkeys, 12 of them were successfully modeled, as confirmed by the imaging findings and staining assessment. One monkey died of brain hernia resulted from intracranial hemorrhage confirmed by necropsy. DSA, CTA, and MRA indicated the presence of an arterial occlusion. CTP and MRI showed acute focal cerebral ischemia. TTC staining revealed infarct lesions formed in the brain tissues.

**Conclusion:**

Our study may provide an optimal non-human primate model for an in-depth study of the pathogenesis and treatment of focal cerebral ischemia.

## Introduction

Stroke is responsible for almost 6 million deaths annually, accounting for more than 10% of all mortalities, and the two-thirds of stroke survivors remain disabled (1). Ischemic stroke occurs when blood flow to the brain is severely interrupted by a thrombus or embolus occluding a cerebral artery, resulting in hypoxic-ischemic necrosis of the corresponding brain tissue, and brain cell death (2–4). The key treatment lies in restoring the blood flow as early as possible, improving brain tissue oxygen supply, saving the ischemic penumbra area, and reducing neuron functional damage (4, 5). Among them, intravenous thrombolysis is one of the main treatment options for ischemia stroke in clinical practice (6–9). To investigate the underlying mechanisms and to develop effective new drugs, a reliable animal model that mimics most of the features of stroke is required.

To date, several reproducible animal models of permanent and transient focal cerebral ischemia have been established, in which a brain-supplying artery is occluded by mechanical devices, such as sutures, clips, and hooks, pharmacological agents, or delivery of blot clots (10–14). However, many of these models poorly mimic clinical thrombus formation, decreasing their clinical utility. The middle cerebral artery (MCA), branching directly from the internal carotid artery (ICA), is the easiest path for thromboembolism and the most common artery involved in a cerebral infarction (15). For studying the consequences of cerebral thromboembolism and evaluating the effects of thrombolytics, animal models, in which MCA or MCA branches were occluded by the autologous blood clots of defined length and diameter, have been established (9, 16–19). However, these models were mainly in rats and mice, with great genetic and epigenetic differences between them and humans (18, 20). Therefore, an animal model based on the adult cynomolgus monkey, which most closely resembles the human brain regarding to cortical and subcortical anatomy, might be a better option.

In this study, we successfully established an animal model of middle cerebral artery occlusion (MCAO) in cynomolgus monkeys under the guidance of digital subtraction angiography (DSA). Then the clots were dissolved by thrombolytic drugs, namely, alteplase (rt-PA), and assessed with imaging analyses and laboratory tests. Our study may provide an optimal non-human primate model for an in-depth study of the pathogenesis and treatment of focal cerebral ischemia.

## Materials and Methods

### Animals

A total of 15 male cynomolgus monkeys (5–8 years old, weighing 4–8 kg) were purchased from Suzhou Xishan Zhongke Laboratory Animal Co.,Ltd (Suzhou, China). All experiments were approved by the Animal Experiment Ethics Committee of Prisys Biotechnologies Co., Ltd (Shanghai, China) (Approval No. IACUC-2021003) and raised according to the ARRIVE guidelines 2.0 (https://www.arriveguidelines.org) (21).

Animals were housed individually indoors under a 12- light/dark cycle (light on from 07:00 to 19:00), and at a constant room temperature of 22–24°C. Laboratory diet was provided two times daily, supplemented with fresh fruit and vegetables and drinking water. Every effort was made to ensure that the animals were free from pain and discomfort.

### Experimental Methods

Before the operation, neurological assessments were performed to rule out neurological dysfunction. Head and neck computed tomography angiography (CTA), CT perfusion (CTP), and brain magnetic resonance imaging (MRI) were scanned to exclude cerebral vascular and intracranial lesions.

A Toshiba 640-slice spiral CT scanner was used for one-stop whole-brain dynamic volume CTA-CTP scanning. We started scanning after 10 ml iodixanol (370 mgI/ml) was injected at 3.5 ml/s. A total of 792 slices were obtained, with a slice thickness of 1 mm, tube voltage of 120 KV, and tube current of 225 mA.

An MRI was performed using a Siemens MAGNETOM Skyra 3.0 T scanner (Syngo Via system) with cranial coils. The scanning sequences included diffusion weighted imaging (DWI), apparent diffusion coefficient (ADC), susceptibility weighted imaging (SWI), T2-weighted imaging (T2WI), T2-fluid attenuated inversion recovery (T2-FLAIR), and 3D time of flight-magnetic resonance angiography (3D TOF-MRA). DWI parameters are: repetition time (TR) = 6000 ms, echo time (TE) = 99ms, b = 1000s/mm^2^, slice thickness = 3.0 mm, field of view (FOV) = 22.5 cm × 22.5 cm, matrix = 192 × 225. SWI parameters are: TR = 27 ms, TE = 20 ms, slice thickness = 1.0 mm, FOV = 18.1 cm × 20 cm, matrix = 256 × 200. T2WI parameters are: TR = 3,200 ms, TE = 407 ms, slice thickness = 0.86 mm, FOV = 22 cm × 22 cm, matrix = 220 × 256. T2-FLAIR parameters are: TR = 5,000 ms, TE = 386 ms, slice thickness = 0.86 mm, FOV = 16 cm × 16 cm, matrix = 220 × 256. 3D TOF-MRA parameters are: TR = 21 ms, TE = 3.51 ms, slice thickness = 0.35 mm, FOV = 19.9 cm × 22 cm, and matrix = 220 × 384.

#### Preparation of Autologous Thrombosis

**Figure 1:**
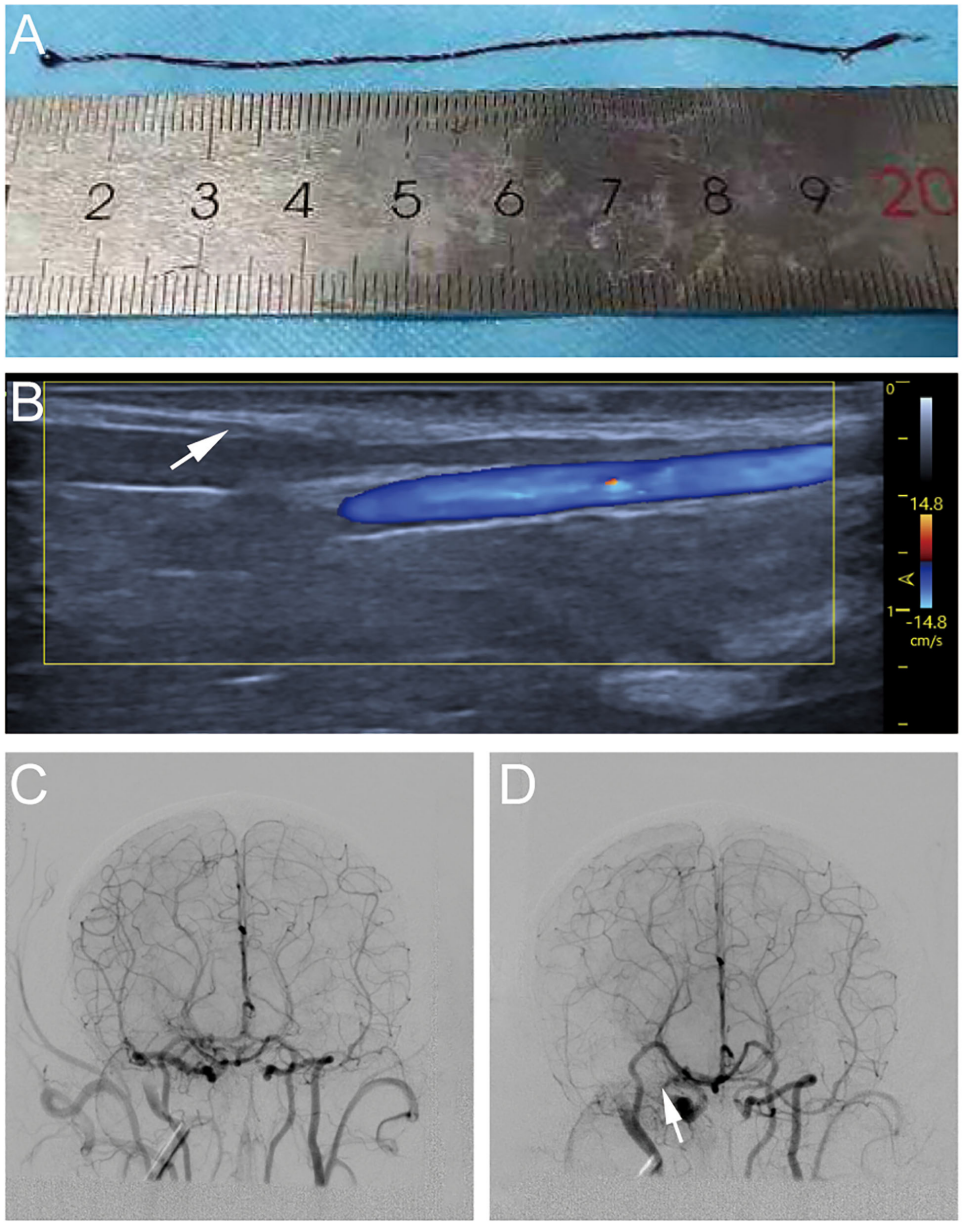
Process diagram of establishing cynomolgus monkey middle cerebral artery occlusion (MCAO) model. **(A)** Autologous venous thrombosis with a diameter of 0.5 mm ofv cynomolgus monkeys required for the experiment. **(B)** A 4F arterial catheter sheath was inserted through the femoral artery under ultrasound guidance. **(C)** Cerebral vascular imaging of cynomolgus monkeys under digital subtraction angiography (DSA) angiography. **(D)** A 5–8 cm thrombi was injected into the internal carotid artery ICA *via* the microcatheter, the right middle cerebral artery (MCA) was not visualized on another angiography.

About 2 ml venous blood was drawn from each monkey 24 h before the operation and rapidly (within 5 min) injected into a silicone tube with a diameter of 0.5 mm. After coagulation, the formed blood clot was placed in saline for repeated rinsing and refrigerated at 4°C (Figure 1A).

#### Ischemia Model

All animals were deprived of food for 24 h and water for 8 h before the operation. Anesthesia was induced with an intramuscular injection of Zoletil and Pentobarbital compound at a dose of 2.5 and 15 mg/kg. After successful anesthesia, all experimental monkeys were obtained about 3–4 ml venous blood for blood biochemical examination.

The animals were immobilized on the operating table in supine position. A 1.7F microcatheter was successfully placed into the MI-segment of right MCA with a 0.014-inch micro-guidewire through a 4F arterial catheter sheath, which was inserted through the femoral artery under ultrasound guidance (Figure 1B). Anteroposterior and lateral MCA angiography was conducted to confirm the correct placement of catheter and to determine the blood flow status as a comparison of the post-occlusion status (Figure 1C). A 5–8 cm thrombi (the exact length depending on the size of the vascular indicated by the MCA angiography) was injected into the ICA *via* the microcatheter. After this, another angiography would be done to confirm that the thrombi completely occluded the MCA (Figure 1D).

#### Thrombolytic Therapy

Thrombolytic therapy with rt-PA was given to 8 of these monkeys at 3 h after the occlusion according to experimental design, and the total administered dose was calculated according to the body weight of the monkeys, with a total administered dose of 1 mg/kg. Approximately 10% of the total dose was dissolved into sterile water to a concentration of 1 mg/ml for injection, and administered by rapid intravenous injection within 1 min. The remaining 90% of the total administered dose was infused within 1 h at a rate of 15–20 drops/min.

#### Imaging Assessment and Blood Test

Imaging examination, such as MRI and MRA, were performed at baseline, 2 h, 5 h, 24 h, and 7 days after induction of ischemic stroke. CTA and CTP were performed at baseline, 24 h and 7 days after the induction of ischemic stroke, and blood examination was performed at the same time after the induction of ischemic stroke.

### Post-Operative Neurological Function Assessment

The behavioral performance of monkeys was continuously recorded for 24 h with a video recorder after operation, and standardized neurological function scores were assessed by two researchers at 6, 12, 24, and 36 h and 2, 3, 4, 5, 6, and 7 days after operation. Of the 100 points possible, 28 were assigned to consciousness, 22 to the sensory system, 32 to the motor system, and 18 to skeletal muscle coordination, as detailed in the Appendix (22).

#### Triphenyltetrazolium Chloride Staining and Infarct Volumes

On the 8th day after operation, the cynomolgus monkeys were autopsied. Brains were rapidly removed within 10 min, and were transferred to phosphate-buffered saline (PBS) solution at 0–4°C, and frozen in a −20°C freezer for 30 min. The cerebellum, olfactory bulb, and lower brainstem were removed. Coronal brain slices were cut to a thickness of 3 mm, and the slices were immersed in a 1% triphenyltetrazolium chloride (TTC) solution in a light-resistant water bath at 37°C for 30 min, and the container was slightly shaken every 5 min to allow adequate staining.

With the use of image analysis software Image J 1.801 (NIH, Bethesda, MD, United States), lesion volumes in each slice were traced and measured by manually outlining the margins of the non-ischemic areas (23, 24). In our study, the lesion volumes in the brain sections were determined by averaging the data obtained by two independent researchers. The lesion volumes were calculated as (S1 + S2 + …… + S10) × 3 mm^3^, where S1–S10 represented slice infarct area in millimeter square. Corrected lesion volumes for edema were calculated with the use of equation: (lesion volume × contralateral volume)/ipsilateral volume (25, 26).

### Statistical Analysis

Normal distribution of neurological deficit scores was checked using Shapiro–Wilk's test for normality. All score data were not normally distributed. Means among different groups were tested using the Mann–Whitney *U*-test, unpaired Student's *t*-test or ANOVA. All statistical analyses were performed with SPSS software (version 26.0, SPSS Inc., Chicago, USA). The value of *p* < 0.05 (two-sided) was statistically significant.

## Results

### General Results of Modeling

Among the 15 cynomolgus monkeys included in the study, 12 were successfully modeled. Among the remaining three monkeys, one was excluded for cerebrovascular malformation; one showed no obvious neurological symptoms after MCAO and no infarction findings on imaging examination; and one died of brain hernia during the experiment. All 12 experimental animals with successful modeling showed different degrees of limb dysfunction after operation, and were divided into two groups, of which eight were experimental groups and underwent rt-PA thrombolytic therapy 3 h after MCAO, and the other four were control groups and given saline after MCAO.

### CTA and CTP Scan

**Figure 2:**
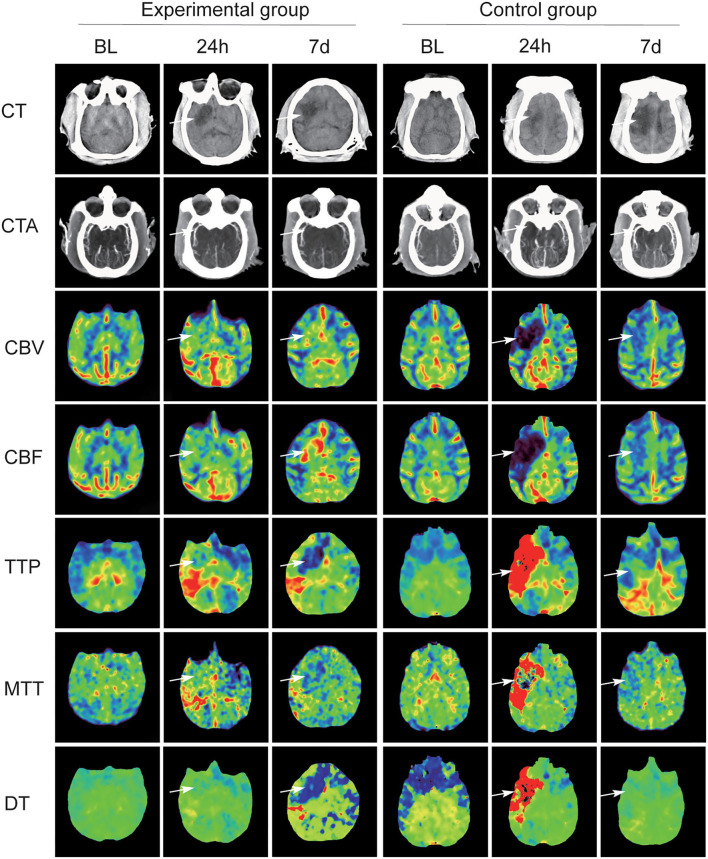
Summary CT scan images maps of two groups with MCAO. In the experimental group, CT angiography (CTA) scan at 24 h and at day 7 after operation confirmed that MCA and its distal branches showed recanalization, but the MCA in the experimental group was not developed. Significant differences between the two groups on CT perfusion (CTP) scans (white arrow).

All 12 cynomolgus monkeys underwent CTA and CTP scan before the operation and 24 h and 7 days after operation. Figure 2 shows the changes of CT scan images of animals. Pre-operative brain CTA showed that MCA and distal branches were clearly visualized in both groups of monkeys, and CTP revealed no abnormalities in brain tissue perfusion.

In the experimental group, CTA scan at 24 h and at day 7 after operation confirmed that MCA and its distal branches showed recanalization, and the number of revealed distal branch vessels gradually increased. CTP scan at 24 h and at day 7 post-MCAO confirmed that the blood supply area of the infarcted vessel had increased cerebral blood flow (CBF) and cerebral blood volume (CBV), decreased time-to-peak (TTP) and delay time (DT), and stable mean transit time (MTT). Low-density shadows were observed in the blood supply area of the infarcted vessel, and the size of low-density lesions was slightly reduced at day 7 compared with that at 24-h post-MCAO.

In the control group, the MCA on the infarcted side was not visualized on CTA scan and the distal branches were slightly visualized at 24 h. At day 7, both sides of the MCAs were visualized and the visualized distal branches on the infarcted side were fewer than the other side. On CTP scan, CBF and CBV in the infarcted vascular supply area were decreased at 24 h and restored at day 7. TTP, MTT, and DT showed a significant increase at 24 h and a decrease at day 7. CT scans at 24 h and day 7 suggested that irregular low-density infarcts appeared in the infarcted vascular supply area, and the size of the lesions was significantly increased.

### MRI and MRA Scan

**Figure 3:**
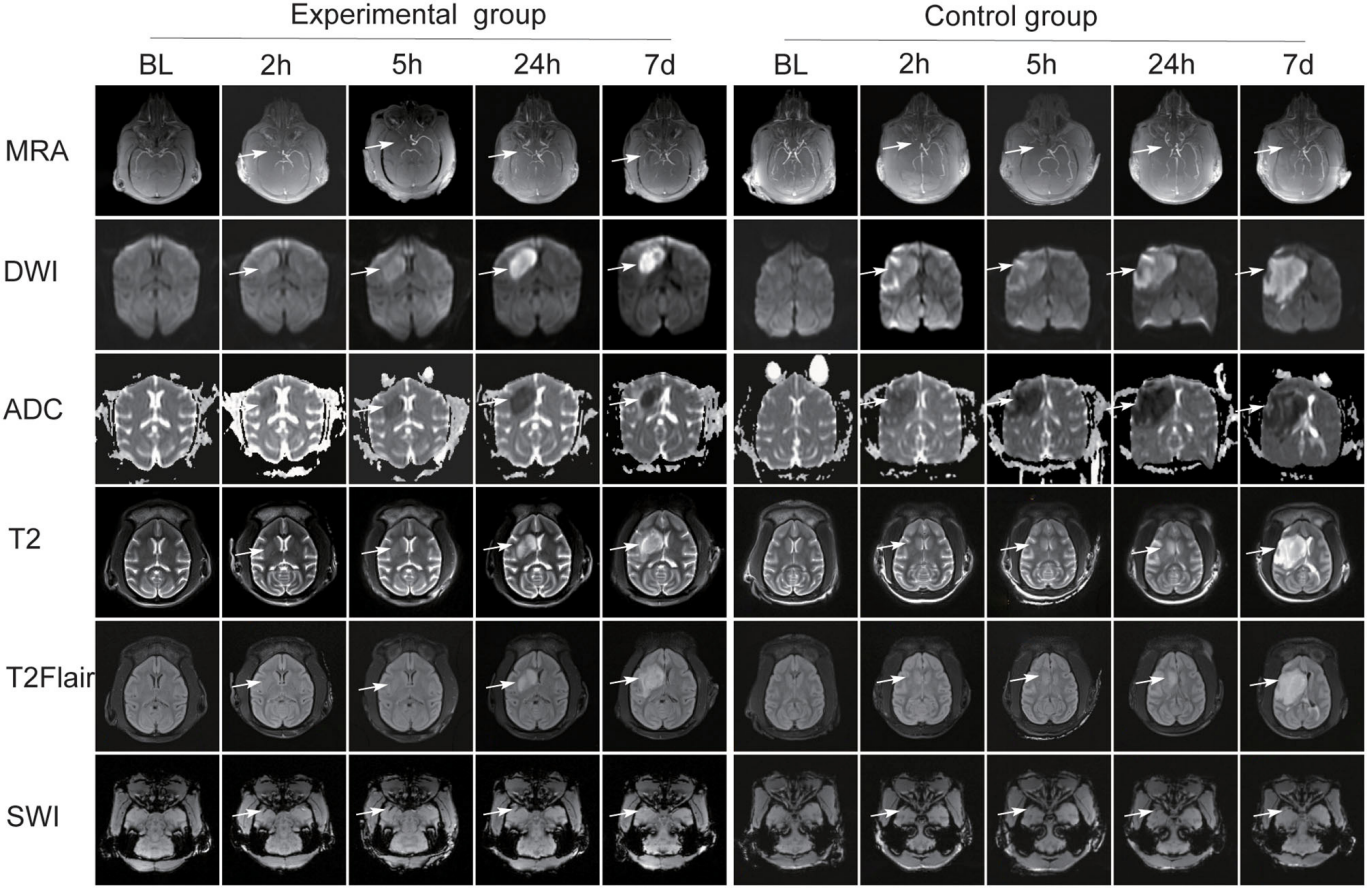
The post-operative MRI image changes in the two groups of animals. Both groups presented different changes over DWI, ADC, SWI, T2, and T2-FLAIR sequences (white arrow). The MCA was absent at 2 h after operation. In the experimental group, MCA reconnected on the infarcted side after thrombolytic therapy. In the control group, brain magnetic resonance angiography (MRA) scans revealed the occluded MCA was absent (white arrow).

The signals on DWI, ADC, SWI, T2WI, and T2-FLAIR sequences were basically the same in all the 12 monkeys before MCAO. The common carotid artery (CCA), external carotid artery (ECA), ICA, anterior cerebral artery (ACA), MCA, posterior cerebral artery (PCA), and circle of Willis were clearly visualized after 3D TOF-MRA reconstruction. Figure 3 shows the post-operative MRI image changes in the two groups of animals.

In both groups, the lesion area showed high signal intensity (SI) on DWI sequence and low SI on ADC maps at 2 and 5 h after operation. In the experimental group, the size and intensity of it increased at 24 h and decreased at day 7, and the pattern was similar on ADC maps. In the control group, the lesion area size and SI gradually increased at 24 h and at day 7. Low SI at corresponding area was shown on ADC maps and the signal gradually decreased at 24 h and at day 7.

In both groups, the signals in T2WI and T2-FLAIR sequences at 2 and 5 h did not change significantly compared with those at baseline, and the size and SI of the lesion area gradually increased at 24 h and day 7.

On SWI, hypointense streaks were observed in the M1-segment of MCAs at 2 and 5 h after operation, with a slightly larger diameter than the artery and no visualization of the distal vessels. No thrombus was observed in the MCA at 24 h and day 7. At 2 and 5 h, multiple thickened venous vascular shadows were observed in the infarct area, which were different from the arterial course and distribution, and multiple small hemorrhagic hypointense foci were observed in the infarct area and adjacent areas. At 24 h and day 7, the thickened venous vascular shadows and hemorrhagic foci were not clearly visualized.

High-resolution craniocerebral artery angiography was obtained by 3DTOF-MRA in both groups. MRA scans revealed that the MCA was absent at 2 h after operation. In the experimental group, MRA scans at 5 h, 24 h, and 7 days after thrombolytic therapy showed MCA recanalization on the infarcted side, representing by a gradual enhancement of vascular SI and an increase in the number of visualized distal branch vessels. In the control group, MRA scans at 5 h, 24 h, and 7 days revealed the occluded MCA was absent.

### Post-Operative Neurological Deficit Score

**Figure 4:**
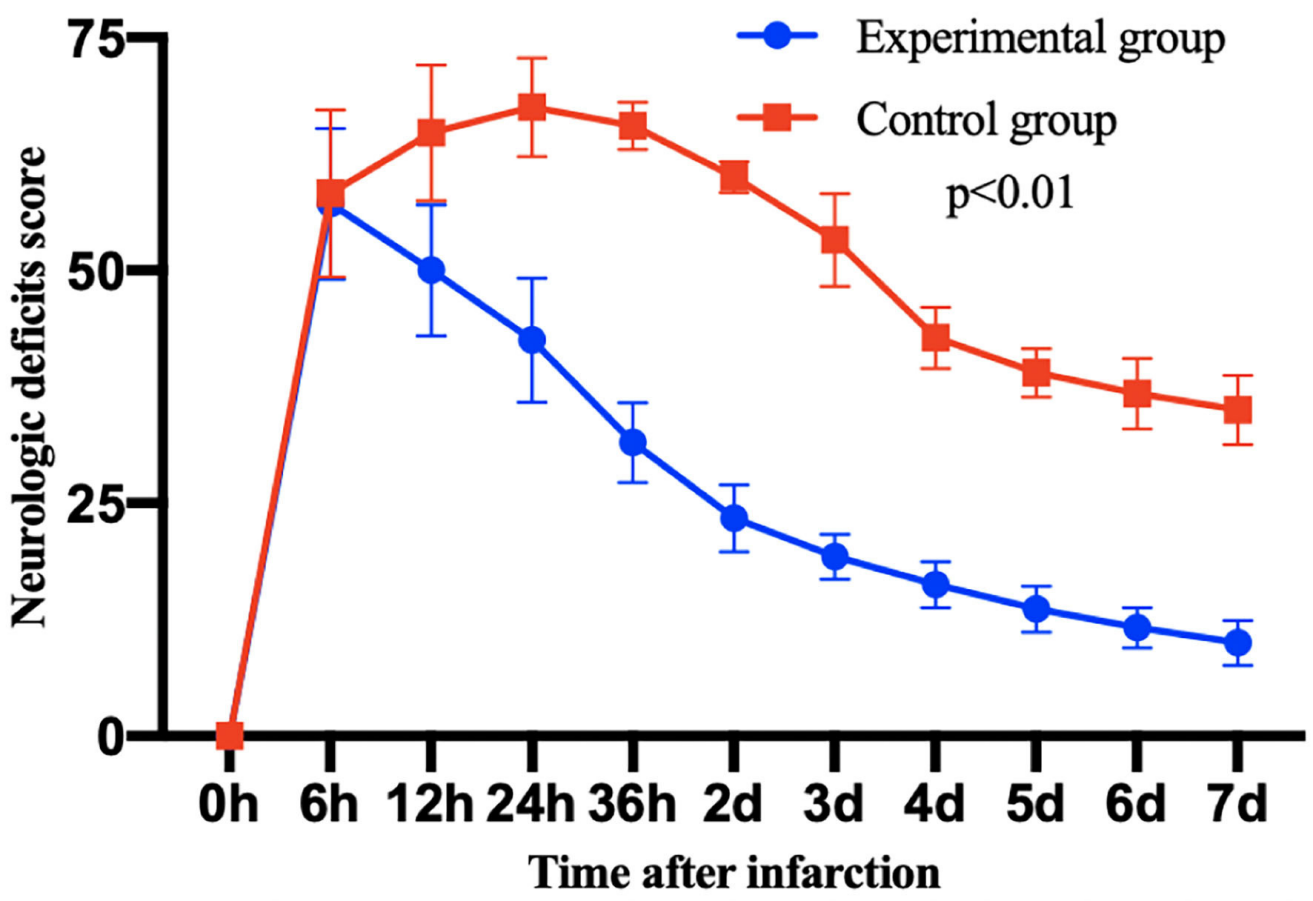
The post-operative neurological deficit score of monkeys in each group after 7 days infarction. Then, 7 days after thrombolytic treatment, the scores of experimental group were significantly decreased compared with the control group. Data are mean ± SD. The *y*-axis represents neurological deficit score, the *x*-axis represents time after infarction.

All the survived 12 monkeys met the inclusion criteria of neurological scoring. The neurological scores of each group are compared in the Figure 4. The neurological scores of both groups increased 6 h after the MCAO, and then the nerve score significantly decreased more after thrombolytic therapy in the experimental group than in the control group.

### TTC Staining Assessment

**Figure 5:**
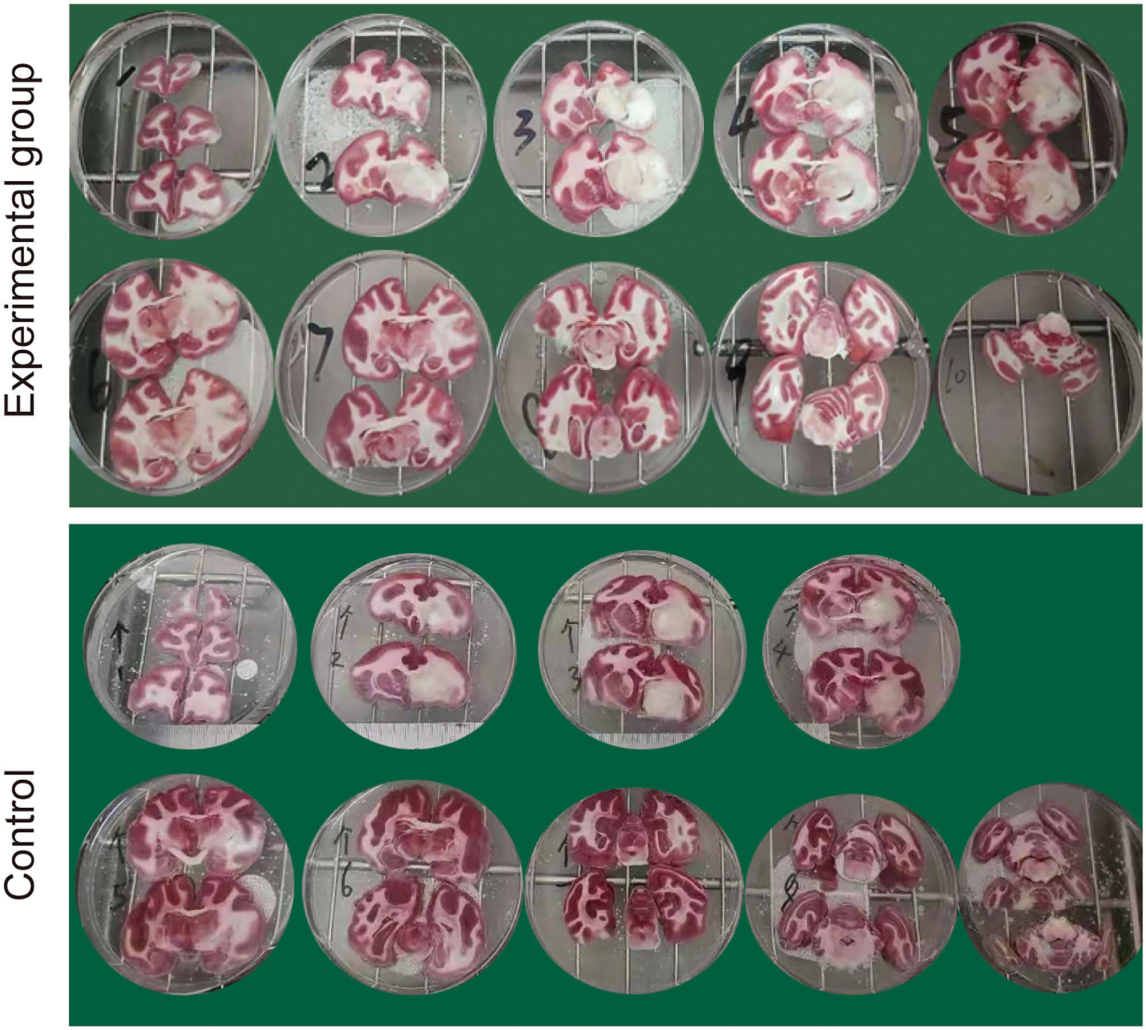
Representative triphenyltetrazolium chloride (TTC) staining of brain sections at 8 days following the MCAO.

The animals were sacrificed on day 8 after occlusion, and brain tissue sections were taken for TTC staining. TTC staining revealed clear delineations between infarcted and normal brain areas and grayish irregular unstained acute ischemic infarct lesions were macroscopically observed in both groups, which were coinciding with the results of MRI scan (Figure 5).

### Infarct Volumes

Infarct volume, such as absolute lesion volume and total brain volume percentage, was measured by using Image J software. The lesion volume in the control group (3.276 cm^3^ ± 0.335) was larger than that in an experimental group (0.934 cm^3^ ± 0.443) (*p* < 0.001, unpaired *t*-test). Consistent with this, the percentage of total brain volume in the control group (10.378 ± 1.707) were larger than that in the experimental group (3.651 ± 0.658) (*p* < 0.001, unpaired *t*-test).

## Discussion

In the present study, the MCAO model induced by autologous clot and rt-PA thrombolysis in cynomolgus monkeys closely simulated the clinical pathogenesis and treatment of ischemic stroke. Among the 15 cynomolgus monkeys, 12 of them were successfully modeled, as confirmed by the imaging findings and staining assessment. This model, featured by an interventional intubation of autologous clot, showed advantages of (1) the most clinically relevant stroke model evoked by autologous blood clots, (2) minimal surgical trauma and complications, (3) high reproducibility and stability with intuitive and accurate positioning of thrombi, (4) abundant and reliable radiographic evaluation, and (5) low-operative mortality.

Stroke is the leading cause of adult disability with the majority caused by brain infarction (27). However, the pathogenesis of stroke has not yet been elucidated. Animal models can contribute to the understanding of the underlying etiology of different subtypes of stroke, leading to better prevention and treatment strategies, and can be used to detect promising therapeutic interventions before trials in human. To date, a large variety of animal models have been established, mainly divided into permanent or transient occlusion (28–31). Permanent ischemia model is widely used in the research of post-ischemic tissue injury, neovascularization, and other aspects, and the temporary ischemia model is suitable for the study of post-ischemic reperfusion. The most common stroke models included intraluminal suture craniectomy, photothrombosis, endothelin-1, embolic stroke model, and so on (32–35). The MCA and its branches are the cerebral vessels that are most often affected in human ischemic stroke, accounting for ~70% of infarcts (16, 36, 37). Thus, techniques that occlude this artery are closest to human ischemic stroke. In addition, using autologous blood clots allows for studying thrombolytic drugs. However, the previous animal models of MCAO have shown various shortcomings, mainly such as large trauma, high animal mortality, uncertainty of embolus size, travel path, and final retention position and inability to effectively evaluate the long-term neurological function of model animals (14, 17, 20, 38–40). In this study, based on the vascular diameter detected with imaging, each thrombus was microscopically divided into emboli of an appropriate size. Autologous clot guided by DSA was accurately localized in the target vessel, with minor trauma, the high success rate of modeling, and the quantifiable records of lesions in the target vessel supply area.

Most stroke experiments are performed in small animals (e.g., mice, rats, and rabbits) (14). The use of small animals has obvious advantages—lower cost and greater acceptability from an ethical perspective—compared with larger animals (41). However, there are important differences between them and human, such as brain size, length, and the structure of perforating arteries. It was also shown that while in humans the percentage of white matter accounts for 60% and about 40% in monkeys, it decreases to 20% in rabbits, 15% in rats, and as low as 10% in mice (42). Thus, an animal model based on cynomolgus monkeys, which has 90–93% genetic similarity with humans, might be a better option (43). Moreover, compared with small animals, monkeys have more operating space and intuitive experimental results.

Ischemic stroke is a highly complex and heterogeneous disorder due to various factors, such as the duration and severity of ischemia, the existence of collateral circulation, and the localization of the infarct (44). It is particularly important to use imaging tools to assess and quantify the severity of acute ischemic stroke, and to predict and evaluate the efficacy. The DWI sequence of MRI is currently widely used in the diagnosis and treatment of hyperacute and acute ischemic stroke and is the most sensitive and accurate imaging examination for an early diagnosis of cerebral infarction. CTP can accurately determine the hemodynamic changes of brain tissue at different stages after ischemia. CTP combined with dynamic CTA can show the blood flow and provide more accurate imaging evidence for clinical practice (9, 45–49). Therefore, in this study, CTA, CTP, and MRI scans were performed on the animals at different stages after model establishment, and the diagnostic value of the above imaging tools in the infarction model was assessed using DSA results as a reference.

Our study has some limitations. First, the experimental duration of the study is too short to conduct a sequential study on the animal model, and key data, such as the recovery of long-term neurological function after infarction and the cognitive status of the animals have not been effectively collected. Second, ligating the distal femoral artery usually took about 30 min, which may cause lower limb dysfunction, and then affect the neurological function score. Third, stroke mainly affects older adult patients exhibiting vascular risk factors and comorbidities, thus healthy cynomolgus monkeys with autologous thrombus could not completely mimic the pathological process of stroke. In our study, the infarcted vessels in the control group showed autolysis at day 7, which was related to the better vascular conditions and physiological response of the animals.

## Conclusions

In conclusion, the model of MCAO in cynomolgus monkeys, established by introducing autologous venous thrombosis into MCA guided by DSA, was found to closely simulate the pathological and therapeutic evolution of ischemic stroke. The technique may serve as an optimal method for the research work of the prevention and treatment strategies of ischemic stroke. However, how neuroimaging can more accurately assess the onset time of stroke, predict the salvageable brain tissue, and determine the population with potential benefits of thrombolytic therapy remains to be further explored.

## Data Availability Statement

The original contributions presented in the study are included in the article/supplementary material, further inquiries can be directed to the corresponding author/s.

## Ethics Statement

The animal study was reviewed and approved by Shanghai-Prisys IACUC.

## Author Contributions

JY, HShe, SC, and ZQ conceived the study, analyzed data, drafted the manuscript, and submitted the manuscript. HSha, HD, YC, LH, FZ, WL, and YW revised the manuscript critically for important intellectual content. All authors read and approved it for publication.

## Conflict of Interest

WL was employed by Prisys Biotechnologies Co., Ltd. The remaining authors declare that the research was conducted in the absence of any commercial or financial relationships that could be construed as a potential conflict of interest. The reviewer J-FL declared a shared affiliation with the authors JY, HSha, HD, YC, LH, FZ, YW, and HShe to the handling editor at the time of review.

## Publisher's Note

All claims expressed in this article are solely those of the authors and do not necessarily represent those of their affiliated organizations, or those of the publisher, the editors and the reviewers. Any product that may be evaluated in this article, or claim that may be made by its manufacturer, is not guaranteed or endorsed by the publisher.
